# The Nrf2 inhibitor brusatol synergistically enhances the cytotoxic effect of lapatinib in HER2-positive cancers

**DOI:** 10.1016/j.heliyon.2022.e10410

**Published:** 2022-08-29

**Authors:** Ziyin Tian, Yan Yang, He Wu, Yongye Chen, Hao Jia, Lei Zhu, Runjia He, Yibo Jin, Bei Zhou, Chunpo Ge, Yanxia Sun, Yun Yang

**Affiliations:** a School of Basic Medical Sciences, Xinxiang Medical University, Xinxiang, China; b Henan International Joint Laboratory of Immunity and Targeted Therapy for Liver-intestinal Tumors, Xinxiang, China; c Department of Nucleus Radiation-related Injury Treatment, PLA Rocket Force Characteristic Medical Center, Beijing, China; d Department of Galactophore, The First People's Hospital of Xinxiang, Xinxiang, China

**Keywords:** Nrf2, Brusatol, Lapatinib, HER2, ROS, ERK1/2, AKT

## Abstract

The dual tyrosine kinase (EGFR/HER2) inhibitor lapatinib is currently used to clinically treat HER2-positive breast cancer. However, a majority of patients do not respond to lapatinib therapy within 6 months. Therefore, potentiating the anti-tumor effect of lapatinib by combination treatment has a great potential to overcome the obstacle. Herein, we aim to investigate the anti-tumor activity of lapatinib in combination with brusatol and explore the potential mechanism involved in the combinatorial treatment. Our findings revealed that the Nrf2 inhibitor brusatol potently enhanced the anti-tumor effect of lapatinib against SK-BR-3, SK-OV-3 and AU565 cancer cells in a synergistic manner. Furthermore, we found that lapatinib plus brusatol more effectively decreased Nrf2 level and induced ROS generation in both SK-BR-3 and SK-OV-3 cells. Moreover, we also observed a significant reduction on the phosphorylation of HER2, EGFR, AKT and ERK1/2 in SK-BR-3 and SK-OV-3 cells when treated with lapatinib plus brusatol compared to either agent alone. More importantly, brusatol significantly augmented the anti-tumor effects of lapatinib in the SK-OV-3 xenograft model. In summary, these data provide a potential rationale for the combination of brusatol and lapatinib on the treatment of HER2-positive cancers.

## 1 Introduction

Nuclear factor erythroid 2-related factor 2 (Nrf2) is an important transcription factor regulating anti-oxidant response element (ARE), thereby limiting the production of reactive oxygen species (ROS) to maintain cellular redox status [1]. Nrf2 overexpression is always tightly associated with tumor invasiveness, metastasis, chemotherapy resistance and poor clinical outcomes in many cancer patients [2].

Brusatol is extracted from the seeds of Brucea javanica, which exhibits the potent inhibitory activity against Nrf2 transcriptional signature [3, 4, 5]. Ren et al elucidated that brusatol exerted synergistic anti-tumor effects with cisplatin, a first-line chemotherapeutic for advanced non-smallcell lung cancer (NSCLC) in the A549 cancer cell line [3]. Xiang et al reported that brusatol potentiated gemcitabine-induced apoptosis and growth inhibition in pancreatic cancer cells *in vitro* and *in vivo* [6]. Recently, we also disclosed that trastuzumab markedly enhanced brusatol-induced ROS accumulation and apoptosis level, which resulted in markedly greater anti-tumor effects [7]. Collectively, brusatol has a great potential to be an adjuvant drug in combination with other molecular targeted drugs in treating cancers.

Overexpression of human epidermal growth factor receptor 2 (HER2) was observed in approximately 20% of breast and gastric cancers [8]. Interference with the phosphorylation of HER2 will lead to regression of several downstream signaling cascade(s), such as PI3K/AKT and mitogen-activated protein (MAPK) signaling [9]. Lapatinib was approved by the US Food and Drug Administration (FDA) in 2007 as a dual-tyrosine kinase inhibitor against HER2 and epidermal growth factor receptor (EGFR) that functions by blocking the intracellular ATP binding site of the tyrosine kinase domain and inhibiting the phosphorylation of EGFR and HER2 [10]. In clinical trials, lapatinib has shown relatively weaker cardiotoxicity compared to trastuzumab, the other FDA-approved HER2-targeted therapeutic [11]. However, primary and acquired resistance also limits the clinical application of lapatinib [12, 13, 14, 15]. Collectively, it is necessary to test the new therapeutic strategies for enhancing the efficacy of lapatinib and improving the survival of breast cancer patients.

## 2 Materials and methods

### 2.1 Cell lines and reagents

The human breast cancer cell line AU565, SK-BR-3, ovarian cancer cell line SK-OV-3 and MCF-10A mammary epithelial cells were purchased from the American Type Culture Collection (ATCC). Cells were cultured in Dulbecco's Modified Eagle's Medium (DMEM) (cat. no. SH30022.01, Hyclone, USA), which was supplemented with 4.5 g/L glucose and 10% fetal bovine serum. Lapatinib was purchased from Rhawn. Inc (Shanghai, China). Brusatol was purchased from Yuanye Biotech Corporation (Shanghai, China). It is over 95% pure determined by HPLC. The stock solution of brusatol was prepared by dissolving in DMEM with 0.25% dimethyl sulfoxide (DMSO). N-acetyl-L-cysteine (NAC) was purchased from Sigma-Aldrich. Inc (Mo, USA). The antibodies were utilized as following: Nrf2 (1:1000; cat. no. 16396-1-AP; ProteinTech Group, Inc.), HO-1 (1:1000; cat. no. 10701-1-AP; ProteinTech Group, Inc.), HER2 (1:1,000; cat. no. 54359; Cell Signaling Technology, Inc.), phosphorylated (p)-HER2 (Tyr 1221/1222) (1:1,000; cat. no. 2243; Cell Signaling Technology, Inc.), EGFR (1:1,000; cat. no. 54359; Cell Signaling Technology, Inc.), phosphorylated (p)-EGFR (Tyr1068) (1:1,000; cat. no. 2234; Cell Signaling Technology, Inc.), AKT (1:1,000; cat. no. 10176-2-AP; ProteinTech Group, Inc.), p-AKT (Ser 473) (1:2,000; cat. no. 4060; Cell Signaling Technology, Inc.), ERK1/2 (1:2,000; cat. no. 9102; Cell Signaling Technology, Inc.), p-ERK1/2 (Thr202/Tyr204) (1:2,000; cat. no. 8544; Cell Signaling Technology, Inc.), β-actin (1:5,000; cat. no. ab179467; Abcam) and horseradish peroxidase-conjugated goat anti-mouse/rabbit secondary antibodies (1:5,000; cat. no. SA00001-1 or SA00001-2; ProteinTech Group, Inc.).

### 2.2 Animals

Six-week female BALB/c nude mice (n = 24) (Vital River Laboratory Animal Technology, Beijing, China) were maintained in a sterile environment. The animal research was conducted according to the Principle of Laboratory Animal Care (NIH Publication No. 85–23, revised in 1985). All experimental protocols were performed under the approval of the Animal Experimentation Ethics Committee of Xinxiang Medical University. Animals were treated in accordance with the guideline of the Animal Care and Use Committee of Xinxiang Medical University.

### *2.3* *In vitro* cytotoxicity assay

Cells (SK-BR-3, AU-565 and SK-OV-3) were plated at a density of 5×10^3^ cells per well in 96-well plates and incubated with lapatinib, brusatol or lapatinib in combination with brusatol for 48 h. Cell viability was then determined by cell counting kit-8 (Dojindo) based on absorbance at 450 nm. The percentage of surviving cells was calculated using the following formula [(A450 of experiment – A450 of background)/(A450 of control – A450 of background)] × 100. Combination index (CI) values were calculated using the Chou-Talalay method by Compusyn Software. Drug synergy, addition, and antagonism are defined by C.I. values less than 1.0, equal to 1.0, or greater than 1.0, respectively.

### 2.4 Immunoblotting

Cells were lysed in RIPA lysis buffer supplemented with 2 μL/mL protease inhibitor cocktail (Sigma) and 10 μL/mL phosphatase inhibitor cocktail (Sigma) for 10 min at 4 °C. The protein concentration of the supernatants was measured by a QuantiPro BCA protein assay kit (Sigma Aldrich). After denaturation, total cell lysates were separated using SDS–PAGE and immunoblotted with primary antibodies and HRP-conjugated secondary antibody as mentioned above. After wash of the membrane, image acquisition was performed using an ChemiDoc imaging system (Bio-Rad Laboratories, Inc.) with the sensitive ECL reagent (Millipore).

### 2.5 Reactive oxygen species (ROS) detection

The level of intracellular reactive oxygen species was routinely detected with 2′, 7’ - dichlorodihydrofluorescein diacetate (DCFH-DA) (Sigma Aldrich) according to the manufacturer's protocol. Briefly, the cells (1×10^5^/well) were treated with the presence or absence of phosphate buffer saline (PBS), brusatol, lapatinib or NAC at 37 °C for 6 h. Then, DCFH-DA (10 μM) diluted by serum-free medium was added to the cells. After incubation for 30 min, the cells were washed 3 times with PBS and collected. The relative levels of fluorescene were recorded by flow cytometer (BD Biosciences) using excitation and emission spectra of 488/525nm.

### 2.6 Apoptosis analysis

Cells were seeded in 6-well plates at a density of 2×10^6^/well and exposed to different drugs. After incubation for the indicated time, the cells were treated with 0.25% trypsin without EDTA, and incubated with Annexin V and propidium iodide (PI) (Dojindo) for 15 min at room temperature in the dark. The rate of apoptotic cells was determined by flow cytometer (BD Biosciences) and analyzed by FlowJo software.

### 2.7 Lipid peroxidation assay

The commercial MDA detection kit (Beyotime, China) was used to quantify the generation of malondialdehyde (MDA) according to the manufacturer's protocol. In brief, cells were plated at a density of 1×10^6^ cells per well in 6-well plates and incubated with lapatinib, brusatol or lapatinib in combination with brusatol for the indicated time. Then, cells were lysed in RIPA lysis buffer. The protein concentration of the total cell lysis was measured by a QuantiPro BCA protein assay kit (Sigma Aldrich). The level of MDA was presented as nmol/mg protein.

### 2.8 Transfection with small interfering RNA (siRNA)

The small interfering RNA (siRNA) against human Nrf2 and scramble siRNA were synthesized by RiboBio Inc (China). The target siRNA sequences directed against human Nrf2 were 5′-GAGAAAGAAUUGCCUGUAA-3′ (siRNA-02) and 5′-GCAACAGGACAUUGAGCAA-3′ (siRNA-03), respectively. Cells were transfected with siRNA using Lipofectamine 3000 (Invitrogen, USA) according to the manufacturer's instructions. The final concentration of the siRNAs was 20 nmol/L.

### 2.9 *In vivo* therapy study

SK-OV-3 cells (1 × 10^7^ per mouse) mixed with PBS were inoculated subcutaneously into the right flank of nude mice. The mice were randomly divided into 4 groups of 6 mice each when tumor volumes reached an average of about 100 mm^3^. Mice were intraperitoneally injected with PBS or brusatol (2 mg/kg) once per day for 18 days. We treated mice with lapatinib (100 mg/kg) daily via oral gavage. Tumors were measured with digital calipers, and tumor volumes were calculated by the formula: Volume = [Length × (Width)^2^]/2. Mice were euthanized with CO_2_ asphyxiation.

### 2.10 Statistical analysis

Statistical analysis was performed with the Graphpad Prism version 5.0 (Graphpad software). Data are shown as mean ± SD. Statistical analysis was performed by Student's unpaired t test to identify significant differences unless otherwise indicated. Nonlinear regression analyses were used to fit curves.

## 3 Results

### 3.1 Brusatol synergistically enhanced growth inhibition caused by lapatinib in SK-BR-3, SK-OV-3 and AU565 cells

**Figure 1:**
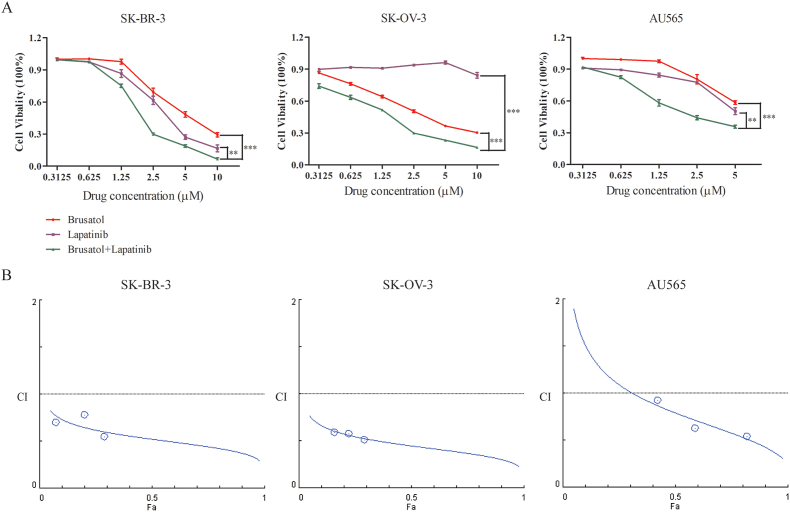
Brusatol in combination with lapatinib synergistically inhibited the growth of HER2-overexpressed SK-BR-3, SK-OV-3 and AU565 cells (A) SK-BR-3, SK-OV-3 and AU565 cell lines were treated with brusatol, lapatinib or brusatol plus lapatinib in a dose range for 48 h. CCK-8 assays were used to measure the cell viability. Points, mean of 3 independent CCK-8 assays; Bars, SD. ∗*p* < 0.05, ∗∗*p* < 0.01, ∗∗∗*p* < 0.001 (B) The synergistic effect of lapatinib in combination with brusatol was evaluated on the growth of SK-BR-3, SK-OV-3 and AU565 cell line. Combination index (CI) values were calculated using the Chou-Talalay method. Drug synergy, addition, and antagonism are defined by CI values less than 1.0, equal to 1.0, or greater than 1.0, respectively.

In the previous study, we found that brusatol exerted anti-tumor effects against HER2-overexpressed cancer cells by repressing HER2-AKT/ERK1/2 signaling pathway [7]. Based on these results, we hypothesize that addition of brusatol to lapatinib may result in a therapeutic benefit in treating HER2-positive cancers. Results revealed that lapatinib in combination with brusatol exhibited a significantly enhanced inhibitory activity than that of either agent alone in all three HER2-positive cancer cells including SK-BR-3, SK-OV-3 and AU565 cancer cells (Figure 1A). Moreover, the superior effects of lapatinib plus brusatol was synergistic on SK-BR-3, SK-OV-3 and AU-565 cells by utilizing the method of Chou and Talalay to establish drug C.I. values (Figure 1B). To further validate the potential toxicity of the combinatorial treatment on normal cells, we also examined the cytotoxic effects of brusatol plus lapatinib on normal mammary epithelial cells MCF-10A. Results showed that the combinatorial treatment has no obvious toxic effect on MCF-10A cells (Supplementary Figure S1).

### 3.2 Brusatol sensitizes HER2-positive cells to lapatinib by inducing the up-regulation of the ROS level in SK-BR-3 and SK-OV-3 cancer cells

**Figure 2:**
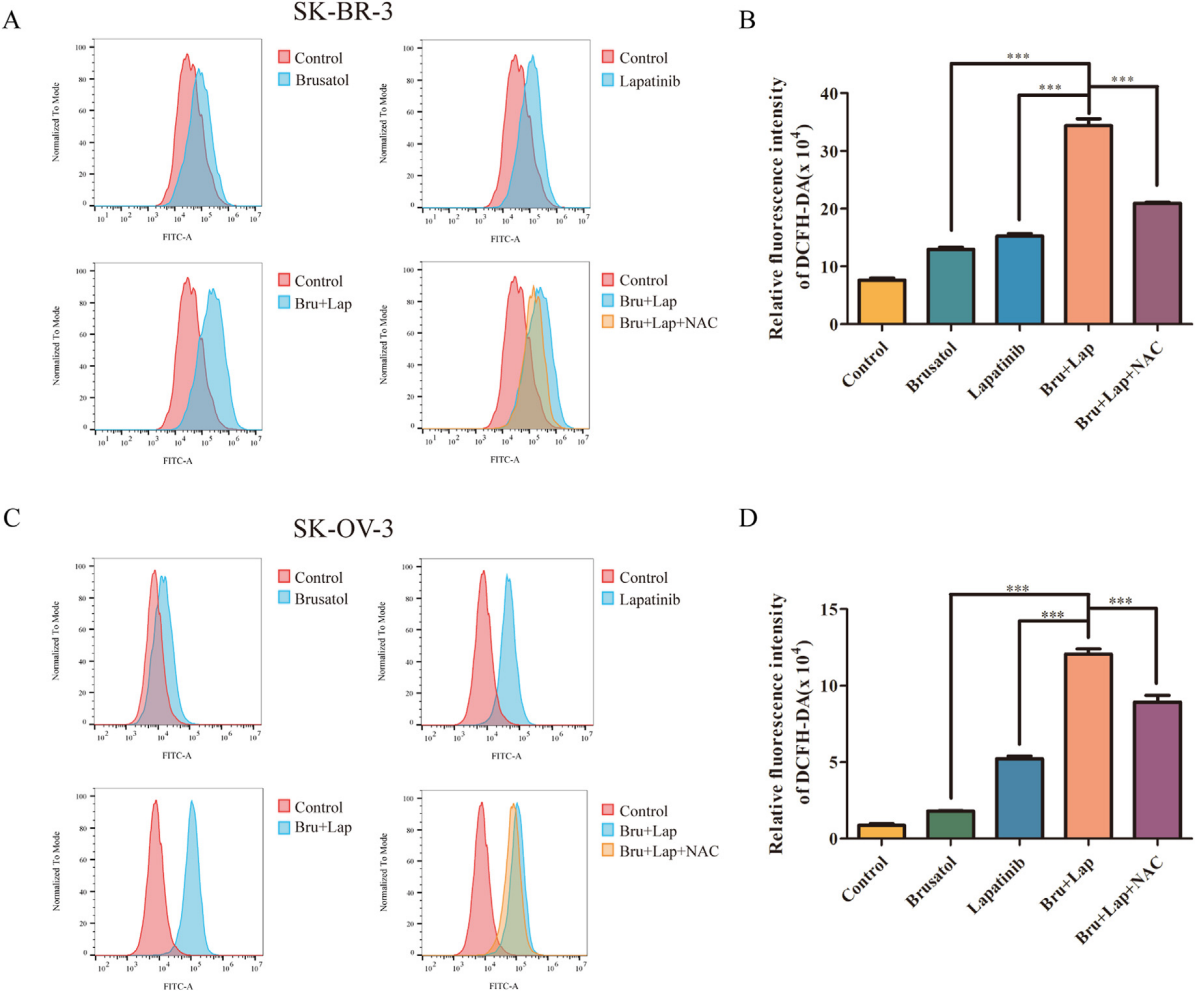
The level of ROS production was observed in SK-BR-3 and SK-OV-3 cells treated with lapatinib plus brusatol (A) SK-BR-3 cells were treated with PBS (Control), lapatinib (1 μM), brusatol (1 μM), lapatinib (1 μM) plus brusatol (1 μM) or lapatinib (1 μM) plus brusatol (1 μM) plus NAC (10 mM) for 6 h, and flow cytometry was used to analyze the level of ROS accumulation in cells after DCFH-DA was added to stain the cells (B) Bar graphic representations of the fluorescence intensity upon different treatments relative to control in SK-BR-3 cell line were shown. Data was presented as mean ± SD. ∗∗∗*p* < 0.001 (C) ROS accumulation was also examined in SK-OV-3 cells. The procedure is the same as that given for (A) in condition (D) Bar graphic representations of the fluorescence intensity upon different treatments relative to control in SK-OV-3 cell line were shown. Data was presented as mean ± SD. ∗∗∗*p* < 0.001.

Recent studies illustrated that brusatol might provide potential clinical benefit especially when combined with anti-tumor drugs that stimulate ROS production [6, 16]. Therefore, we also examined the effects of combinatorial treatment on ROS accumulation utilizing FACS assay. Results revealed that co-treatment of lapatinib with brusatol resulted in a significantly greater increase on ROS production than brusatol or lapatinib treatment alone in both SK-OV-3 and SK-BR-3 cancer cells (Figures 2A-2D). To further provide additional evidence for these results, N-acetyl-L-cysteine (NAC), a type of anti-oxidant agent that can effectively reduce the ROS level was added to SK-OV-3 and SK-BR-3 cells treated with a combination of lapatinib and brusatol. As expected, the co-administration of the anti-oxidant NAC antagonized the elevation in ROS production from both SK-OV-3 and SK-BR-3 cell lines treated with brusatol plus lapatinib (Figures 2A-2D). To conclude, brusatol plus lapatinib was more effective in inducing ROS production than either agent alone, which may explain the superior anti-tumor effects of the combinatorial treatment.

### 3.3 Brusatol in combination with lapatinib potently potentiated cell death in SK-BR-3 and SK-OV-3 cells

**Figure 3:**
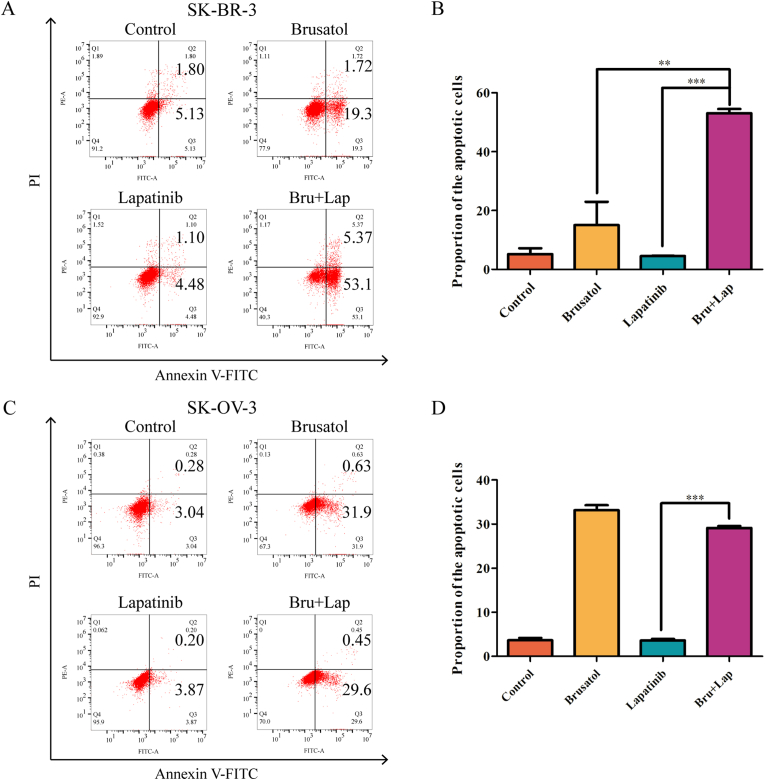
Lapatinib in combination with brusatol potently induced apoptosis in SK-BR-3 cells (A) Induction of apoptosis of SK-BR-3 cells after PBS, brusatol (2 μM), lapatinib (2μM) or the combinatorial treatment for 12 h (B) Apoptosis ratios were measured by flow cytometry. Data was shown with mean ± SD of three independent experiments. ∗∗∗*p* < 0.001 (C) Induction of apoptosis of SK-OV-3 cells after PBS, brusatol (2 μM), lapatinib (2 μM) or the combinatorial treatment for 48 h. Apoptosis ratios were measured by flow cytometry, and flow cytometry was used to analyze the level of ROS in cells after DCFH-DA was added to stain the cells (D) Apoptosis ratios were measured by flow cytometry. Data was shown with mean ± SD of three independent experiments. ∗∗∗*p* < 0.001.

As we know, Nrf2 inhibition by Nrf2-targeted agents renders cancer cells susceptible to cell death [17, 18]. Thus, we evaluated the apoptotic percentage in SK-BR-3 and SK-OV-3 cells when were exposed to brusatol plus lapatinib. Compared to brusatol or lapatinib alone, a combination of brusatol with lapatinib significantly potentiated apoptosis in SK-BR-3 cells (Figure 3A and B). Notably, as shown in Figure 3C and D, combinatorial therapy has not exhibited the superiority in SK-OV-3 cells compared to brusatol group. Ferroptosis is a recently defined form of cell death due to ROS accumulation and lipid peroxidation [19]. Lipid peroxidation test was performed to preliminarily evaluate ferroptosis in both SK-BR-3 and SK-OV-3 cell lines. Our results showed that brusatol treatment alone has not elevated the level of MDA in both SK-BR-3 and SK-OV-3 cancer cells. MDA level was significantly increased in SK-BR-3 cells upon combinatorial treatment compared to that of in lapatinib treatment group, while it was not significantly affected in SK-OV-3 cells treated with lapatinib plus brusatol (Supplementary Figure S2A and B).

Therefore, these results suggested that both apoptosis and ferroptosis may contribute to the synergistic effects in SK-BR-3 cells, while they may be not the main mechanism underlying the superior anti-tumor effects of combinatorial treatment in SK-OV-3 cells.

### 3.4 Lapatinib plus brusatol exhibited a significant effect on regressing Nrf2/HO-1 anti-oxidant and EGFR/HER2-AKT/ERK1/2 signaling pathways in SK-BR-3 and SK-OV-3 cancer cells

**Figure 4:**
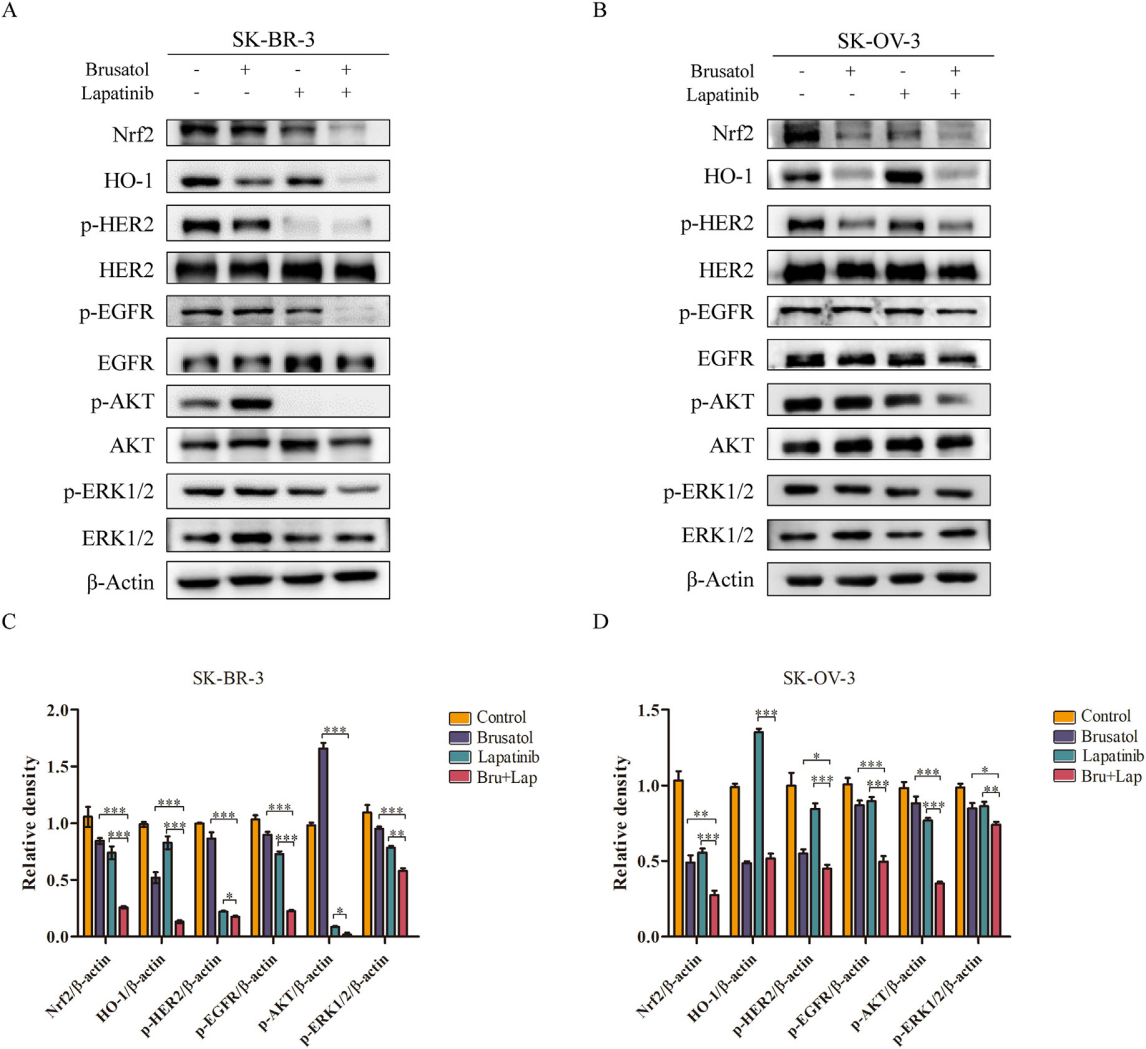
Lapatinib plus brusatol abrogate the activation of Nrf2/HO-1 and EGFR/HER2-AKT/ERK1/2 pathways (A and B) SK-BR-3 and SK-OV-3 cells were treated with lapatinib or brusatol alone, or their combination for 24 h. The changes in Nrf2/HO-1 and EGFR/HER2-AKT/ERK1/2 signaling pathways were monitored by Western Blotting (C and D) Densitometric analysis was performed on the Western Blotting. The levels of Nrf2, HO-1, p-HER2, p-EGFR, p-AKT and p-ERK1/2 were quantified by using the software Image J. The data are expressed as the mean ± SD of three independent experiments. ∗*p* < 0.05, ∗∗*p* < 0.01, ∗∗∗*p* < 0.001. The original blots were provided in supplementary data as Supplementary Figure S4.

To further explore the mechanism involved in synergistic anti-tumor effect, we examined the core protein level involved in Nrf2/HO-1 signaling and EGFR/HER2-AKT/ERK1/2 signaling pathway in both SK-BR-3 and SK-OV-3 cancer cells. Western blot analysis showed that brusatol in combination with lapatinib was more effective in inhibiting phosphorylation of EGFR and HER2 than either single-drug treatment. Moreover, weaker phosphorylation level of AKT and ERK1/2 was observed in the two cell lines treated with contaminant treatment, respectively, compared with single agent treatments (Figures 4A-4D). The similar results were also observed in AU565 cells (Supplementary Figure S3A and B). Thus, these results above suggested that the combinatorial treatment may mainly preclude HER2/EGFR-AKT/ERK1/2 signaling activation, thereby resulted in cell growth repression.

### 3.5 Nrf2 knockdown sensitized SK-OV-3 cells to lapatinib

**Figure 5:**
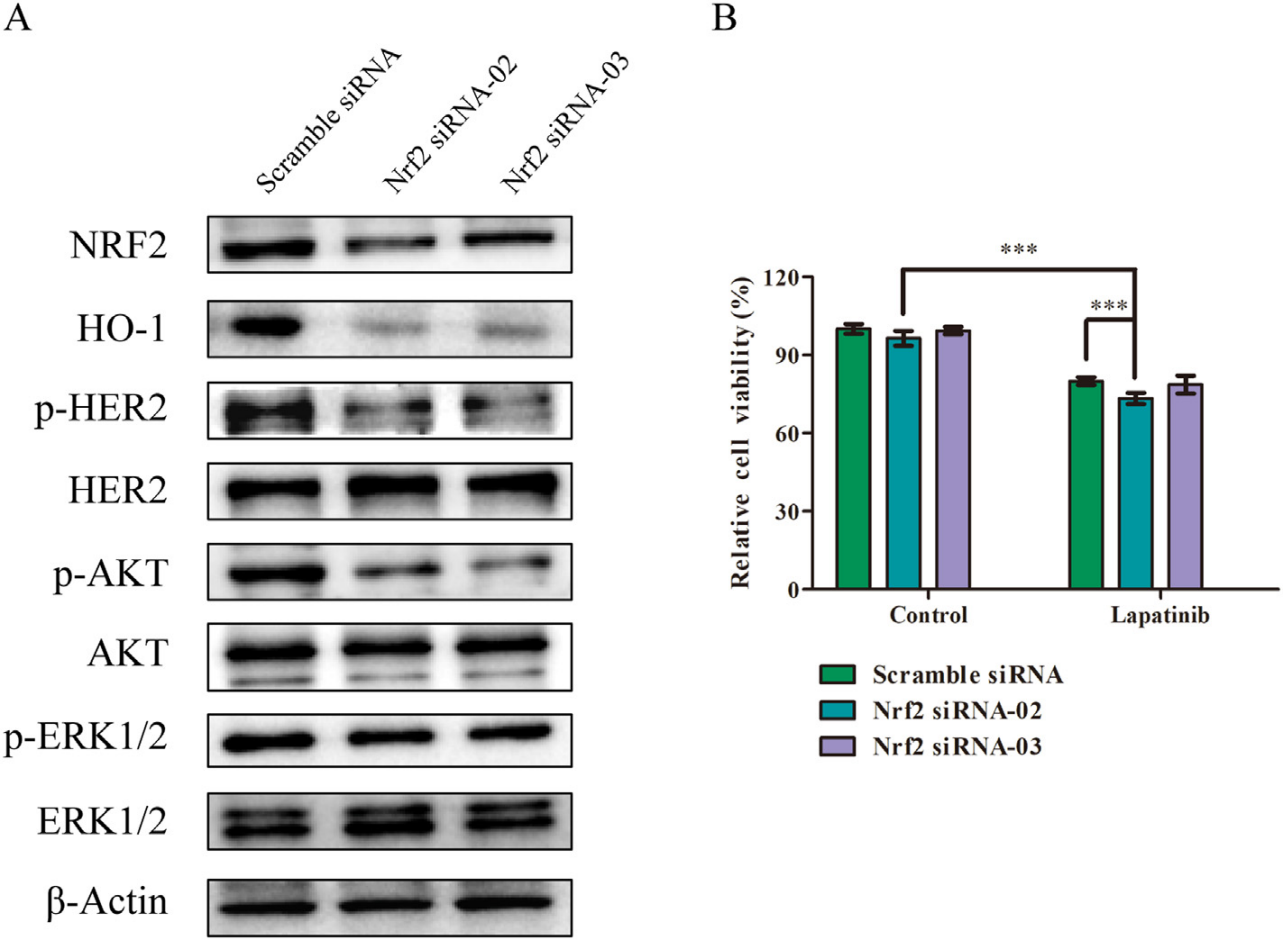
Nrf2 knockdown repressed the activation of HER2 signaling pathway and sensitizes SK-OV-3 cells to lapatinib treatment (A) Effect of Nrf2 knockdown on the expression of HO-1, p-HER2, p-AKT, and p-ERK1/2 were determined after treatment with Nrf2 siRNA or scramble siRNA for 36 h (B) Effect of Nrf2 knockdown on the sensitivity to lapatinib. Cell viability was examined after lapatinib treatment for 36 h in Nrf2 siRNA or scramble siRNA-transfected cells. Cells were transfected with Nrf2 siRNA or scramble siRNA using Lipofectamine 3000 (Invitrogen) according to the supplier's instruction. Data show the mean ± SD (three independent experiments). ∗∗∗*p* < 0.001. The original blots were provided in supplementary data as Supplementary Figure S5.

To further confirm whether brusatol functions by targeting Nrf2, siRNA was transfected to knock down Nrf2 expression in SK-OV-3 cells. Immunoblot analysis showed that Nrf2 knockdown markedly decreased phosphorylation of HER2, AKT and ERK1/2 level in SK-OV-3 cells, which was similar to the effect of brusatol (Figure 5A). Figure 5B showed that Nrf2 silencing in combination with lapatinib was much more potent than either treatment alone in regressing the growth of SK-OV-3 cancer cells. Nrf2 siRNA-transfected SK-OV-3 cells were more sensitive to lapatinib than scramble siRNA-transfected cells. Overall, these results suggested that Nrf2 inhibition increased the sensitivity of SK-OV-3 cells to lapatinib.

### 3.6 Combination therapy of lapatinib and brusatol is superior to single-agent treatment in SK-OV-3 xenografts

**Figure 6:**
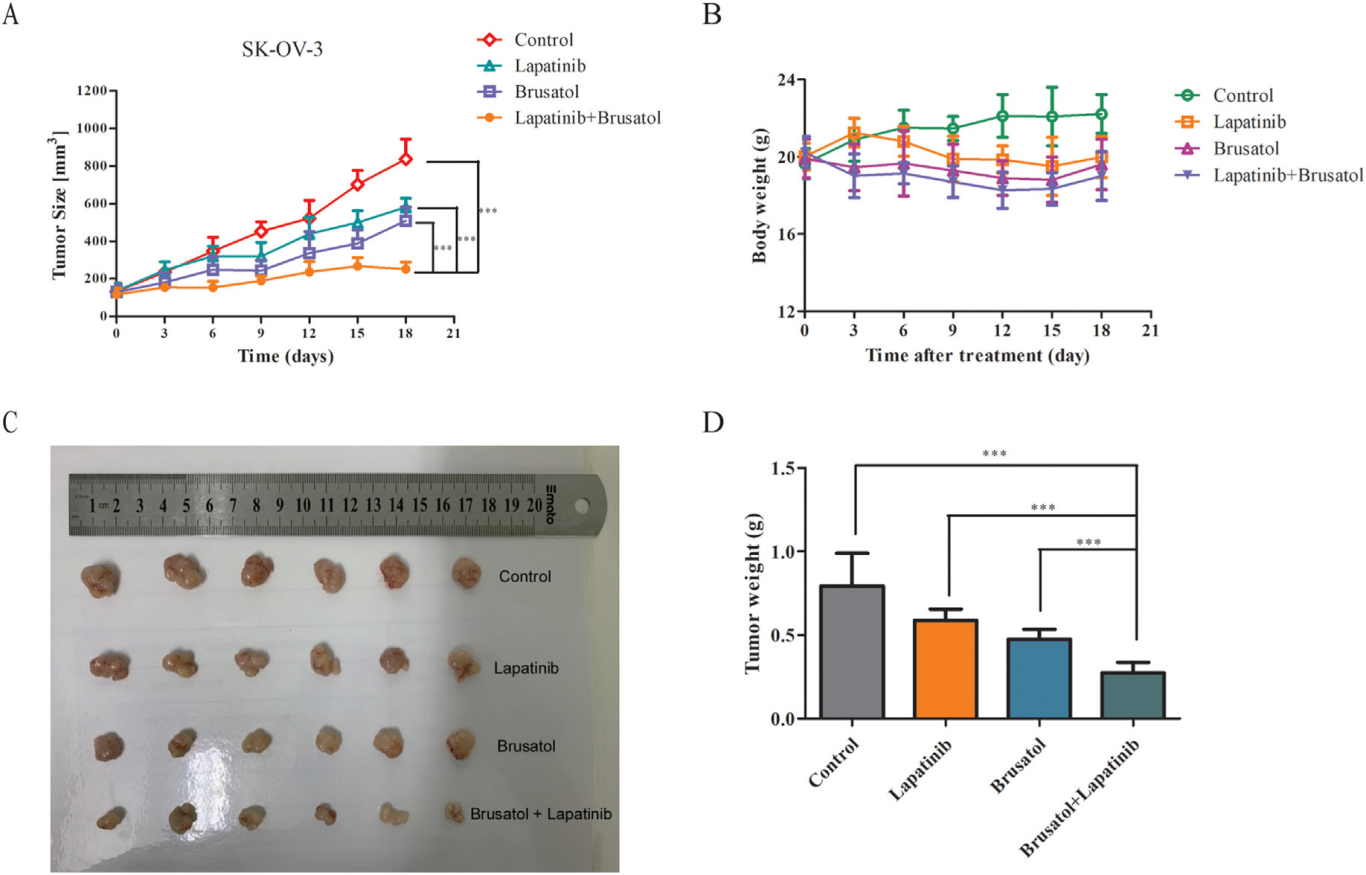
Anti-tumor effects of lapatinib and brusatol alone or in combination in SK-OV-3 tumor xenografts (A) Mean tumor volume of SK-OV-3 xenografts after treated with control (PBS), lapatinib (100 mg/kg), brusatol (2 mg/kg), or lapatinib (100 mg/kg) plus brusatol (2 mg/kg) (B) Mean tumor volume of SK-OV-3 xenografts after injection with control (PBS), lapatinib (100 mg/kg), brusatol (2 mg/kg), or lapatinib (100 mg/kg) plus brusatol (2 mg/kg) (C and D) On day 18 post first injection, xenograft tumors from each group were removed and tumor masses were weighed. Data are shown as mean ± SD (n = 6 mice, each group); ∗*p* < 0.05; ∗∗*p* < 0.01; ∗∗∗*p* < 0.001.

To further evaluate the therapeutic effects *in vivo*, brusatol plus lapatinib was injected in nude mice bearing established SK-OV-3 xenograft tumors. Nude mice bearing SK-OV-3 xenografts were randomized and treated either with lapatinib (100 mg/kg), brusatol (2 mg/kg), or both. Results showing that contaminant treatment of brusatol and lapatinib resulted in a significant benefit over either brusatol or lapatinib alone in SK-OV-3 xenografts (Figure 6A). Meanwhile, we found that the mice in combinatorial group did not significantly lose body weight and behaved normally in the treatment period (Figure 6B). As is shown in Figures 6C and 6D, the treatment with lapatinib and brusatol combination resulted in a 50 % reduction in tumor weight in comparison with control. Besides these, hematoxylin and eosin (H&E) staining also showed that no marked liver toxicity was observed in SK-OV-3 tumor-bearing mice upon treatment with lapatinib plus brusatol (Supplementary Figure S6). Overall, these results above revealed that the combinatorial therapy shows stronger inhibitory effects and appear to be well tolerated in HER2-positive tumor xenografts.

## 4 Discussion

Lapatinib, a dual inhibitor of EGFR and HER2 tyrosine kinase activity, has already been approved by the FDA as a treatment for HER2-positive metastatic breast cancer [20]. Despite the effectiveness, primary and acquired resistance dramatically limits the usage of lapatinib. Therefore, developing a strategy to enhance its therapeutic efficacy is urgent in clinics. Brusatol has been reported to sensitize cancer cells to carboplatin, 5-fluorouracil, gemcitabine, etoposide, and paclitaxel by regressing Nrf2-dependent anti-oxidant response pathway [4, 6, 21]. In the study, we found that brusatol was capable of enhancing the anti-tumor effects of lapatinib in HER2-positive SK-BR-3, AU565 and SK-OV-3 cancer cells in a synergistic manner. Moreover, the synergistic effects were verified in mice bearing SK-OV-3 tumors, which was characterized as a classical model to examine HER2-targeted drugs [22, 23]. Further study will be aimed to explore the effects of lapatinib plus brusatol in lapatinib-resistant cancer cell lines. Overall, our results provide evidence for rational strategies that can promote the efficacy of lapatinib.

It has been previously reported that brusatol treatment rapidly and transiently reduce Nrf2 expression through a posttranscriptional mechanism in Hepa-1c1c7 cells [24]. In our study, we observed the dynamic of Nrf2 inhibition using multiple time points upon brusatol treatment in SK-OV-3 cancer cells. Results showed that the level of Nrf2 was markedly decreased after treatment for 24 h, which suggested that brusatol may function at different time courses among different types of cancer cells (Supplementary Figure S7). Our previous study revealed that brusatol was effective in inhibiting tumor growth and potently enhanced the anti-tumor activity of trastuzumab in HER2-positive cancers [7]. It suggested that dual inhibition of Nrf2 anti-oxidant signaling and HER2-AKT/ERK1/2 signaling is crucial for the anti-tumor effect of HER2-targeted therapeutics in HER2-overexpressed tumors. Therefore, we examined the effects of brusatol plus lapatinib in HER2-positive cancer cells *in vitro* and *in vivo*. As a result, we also observed the synergistic effects of brusatol plus lapatinib in the three cancer cell lines. Until now, resistance to HER2-targeted therapeutics such as lapatinib or trastuzumab during therapy period is still a major obstacle to the successful treatment of breast cancer [25, 26]. Thus, these results provided a potential therapeutic strategy by combining brusatol with other HER2-targeted therapeutics including antibody or small-molecule antagonists to overcome tolerance.

It is well known that the transcription factor Nrf2 always was suppressed by an association with Keap 1 under homeostatic conditions, but it is activated when cells are exposed to oxidative or electrophilic stress. In our study, we observed that the level of Keap1 was not affected upon combinatorial treatment (data not shown). Indeed, Nrf2 can be regulated by several signaling pathways. For example, Rada et al elucidated a Keap1-independent mechanism by which Nrf2 is inhibited that is based on its interaction with the SCF ubiquitin ligase adaptor β-TrCP [27]. Therefore, the detailed mechanism of lapatinib plus brusatol on regulating Nrf2 activity will be further explored in our following study.

**Figure 7:**
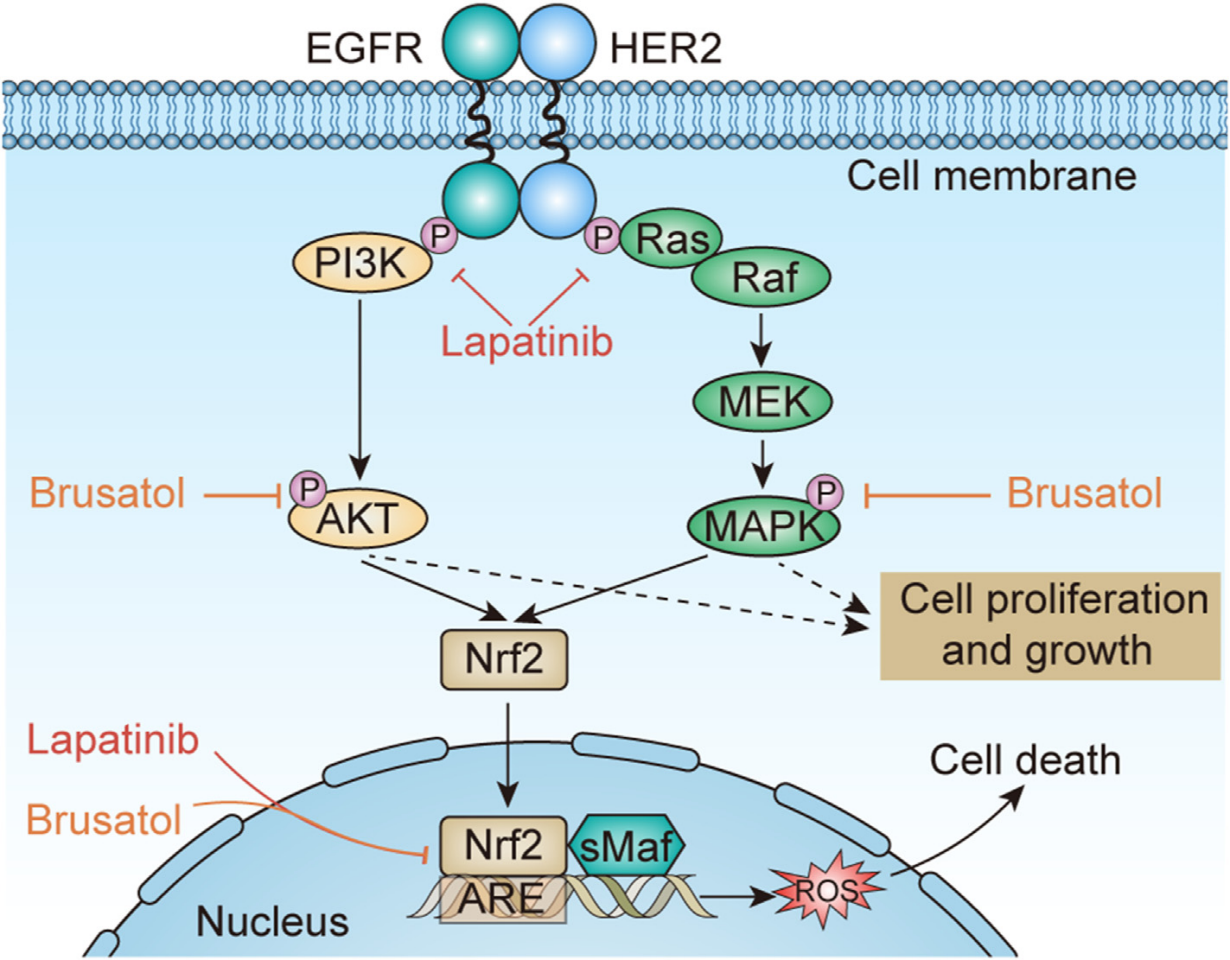
Proposed model of the molecular basis of synergistic interaction between lapatinb and brusatol. Brusatol, in combination with lapatinib, may exert synergistic effects in two ways: (a) combination therapy inhibits the phosphorylation of HER receptors including EGFR and HER2, limiting the activation of their downstream pathways including PI3K-AKT signaling and Ras/Raf/MAPK signaling. (b) combination therapy modulates cell redox homeostasis by decreasing Nrf2 level and preventing the accumulation of Nrf2 in the nucleus, interfering its binding to small Maf oncogene family proteins (Mafs) and antioxidant response elements (AREs) complex, causing the inhibition of antioxidant genes such as heme oxygenase 1 (HO-1) and superoxide dismutase (SOD), thereby resulting in ROS accumulation and cell death.

In the previous studies, modulation of redox homeostasis has been emerged as a new therapeutic strategy in treating solid tumors [28]. According to our findings, we proposed at least 2 possible mechanisms for the synergistic anti-tumor effect of brusatol and lapatinib. The first involves attenuated phosphorylation of HER2 and EGFR and regressed activation of downstream signaling relevant to tumor growth. Additionally, inhibition of Nrf2/HO-1 anti-oxidant signaling and resultant cell death induced by ROS accumulation may also be involved (Figure 7).

## 5 Conclusion

In this study, we investigated the anti-tumor efficacy of Nrf2 inhibitor, brusatol in combination with dual HER2/EGFR inhibitor, lapatinib and explored its potential mechanism of action as a new therapeutic regimen for HER2-positive cancers. These results showed that the combination of brusatol with lapatinib resulted in synergistic anti-tumor effects both *in vitro* and *in vivo*. Thus, the present study provides a potential rationale by combining a novel natural agent brusatol and lapatinib to improve the therapeutic efficacy of lapatinib in treating HER2-positive cancers.

## Declarations

### Author contribution statement

Yun Yang; Yanxia Sun; Ziyin Tian: Conceived and designed the experiments; Performed the experiments; Analyzed and interpreted the data; Contributed reagents, materials, analysis tools or data; Wrote the paper.

Yan Yang; He Wu; Yongye Chen; Hao Jia; Lei Zhu; Runjia He; Yibo Jin; Bei Zhou; Chunpo Ge: Performed the experiments; Analyzed and interpreted the data.

### Funding statement

Professor Yun Yang was supported by the Program for Science＆Technology Innovation Talents in Universities of Henan Province [22HASTIT049].

This work was supported by Natural Science Foundation of Henan (202300410322 and 222300420266); the Training Plan for Young Backbone Teachers in Universities of Henan Province (2020GGJS143); the Key Scientific Research Project of Higher Education of Henan Province (22A310007 and 22B310011); the Science & Technology Project for Young Talents of Henan Province (2020HYTP048 and 2022HYTP042); and the 10.13039/501100013254National College Students Innovation and Entrepreneurship Training Program (202110472008) and Innovation project of Graduate in Xinxiang Medical University (YJSCX202130Y).

### Data availability statement

Data included in article/supp. material/referenced in article.

### Declaration of interest's statement

The authors declare no conflict of interest.

### Additional information

No additional information is available for this paper.

## Appendix A Supplementary data

Supplementary Material-Yun Yang.docx Supplementary Material-Yun Yang.docx
