# Supplementary material for: The Nrf2 inhibitor brusatol synergistically enhances the cytotoxic effect of lapatinib in HER2-positive cancers
Source: Heliyon. 2022 Aug 29;8(8):e10410. doi: 10.1016/j.heliyon.2022.e10410 (PMC9449760; doi:10.1016/j.heliyon.2022.e10410)
Supplement: Supplementary Material-Yun Yang.docx [file mmc1.docx]

# Heliyon

**The Nrf2 inhibitor brusatol synergistically enhances the cytotoxic effect of lapatinib in HER2-positive cancers**

Ziyin Tian^1,#^, Yan Yang^1,#^, He Wu^1,#^, Yongye Chen^1^, Hao Jia^1^, Lei Zhu^3^, Runjia He^1^, Yibo Jin^1^, Bei Zhou^1^, Chunpo Ge^1^, Yanxia Sun^4,*^ and Yun Yang^1,2, *^

^#^These authors have contributed equally to this work and share first authorship

^1^School of Basic Medical Sciences, Xinxiang Medical University, Xinxiang, China;

^2^Henan International Joint Laboratory of Immunity and Targeted Therapy for liver-intestinal Tumors, Xinxiang, China;

^3^Department of Nucleus Radiation-related Injury Treatment, PLA Rocket Force Characteristic Medical Center, Beijing, China.

^4^Department of Galactophore, The First People’s Hospital of Xinxiang, Xinxiang, China;

Correspondence should be addressed to: Yanxia Sun (hn50000@126.com) or Yun Yang (jamesyangyun1@126.com)

**Key words:** Nrf2; Brusatol; Lapatinib; HER2; ROS; ERK1/2; AKT

**Supplementary Figures:**

**
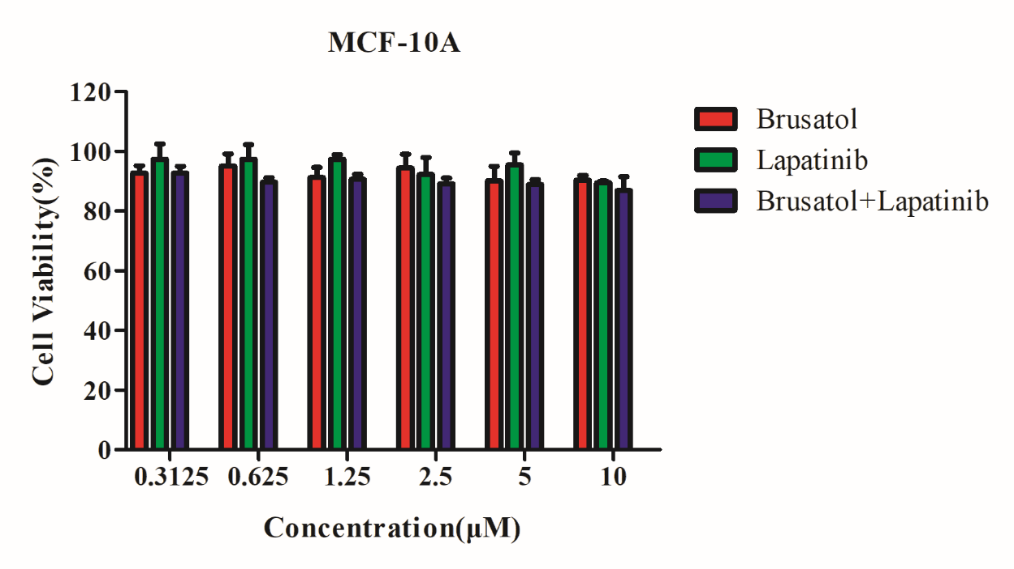
Figure S1 Lapatinib in combination with brusatol has no obvious toxic effect on mammary epithelial MCF-10A cells.** MCF-10A cells were treated with brusatol, lapatinib or brusatol plus lapatinib in a dose range from 0.3125 to 10 μM for 48 h. CCK-8 assays were used to measure the cell viability. Bars, SD.

**
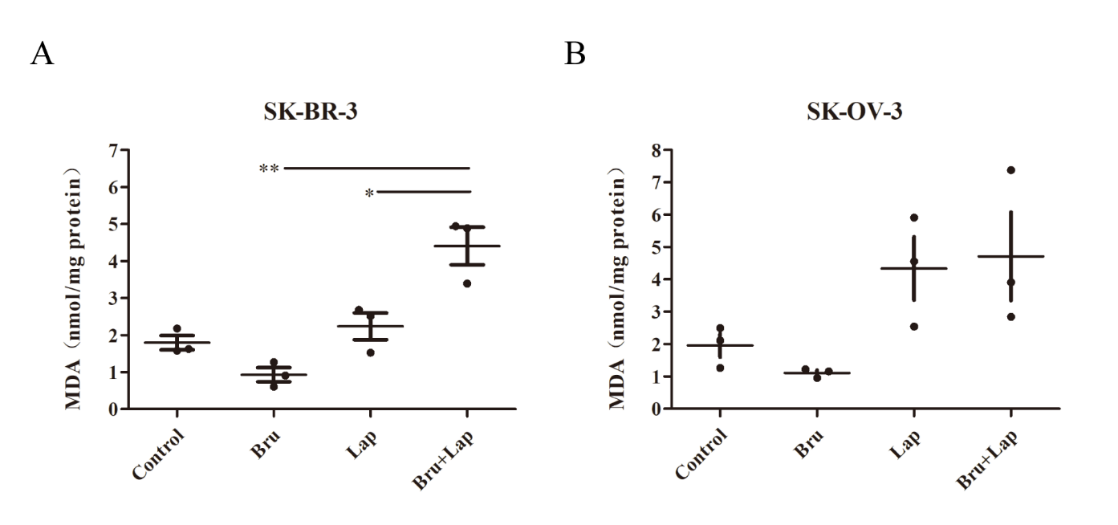
Figure S2 Analysis of MDA level in SK-BR-3 and SK-OV-3 cells upon combinatorial treatment. (A)** SK-BR-3 cells were treated with lapatinib (2 µM) or brusatol (2 µM) alone, or their combination for 12 hours. (B) MDA level was also examined in SK-OV-3 cells upon treatment with lapatinib (2 µM) or brusatol (2 µM) alone, or their combination for 48 hours. * *p* < 0.05, ** *p* < 0.01, *** *p* < 0.001.

**Figure S3 Lapatinib plus brusatol inhibited the activation of** **Nrf2/HO-1 and EGFR/HER2-AKT/ERK1/2 pathways in AU565 cells.**  **(A)** AU565 cells were treated with lapatinib or brusatol alone, or their combination for 24 hours. The changes in Nrf2/HO-1 and EGFR/HER2-AKT/ERK1/2 signaling pathways were monitored by Western Blotting. **(B)** Densitometric analysis was performed on the Western Blotting. The levels of Nrf2, HO-1, p-HER2, p-EGFR, p-AKT and p-ERK1/2 were quantified by using the software Image J. The data are expressed as the mean ± SD of three independent experiments. * *p* < 0.05, ** *p* < 0.01, *** *p* < 0.001.


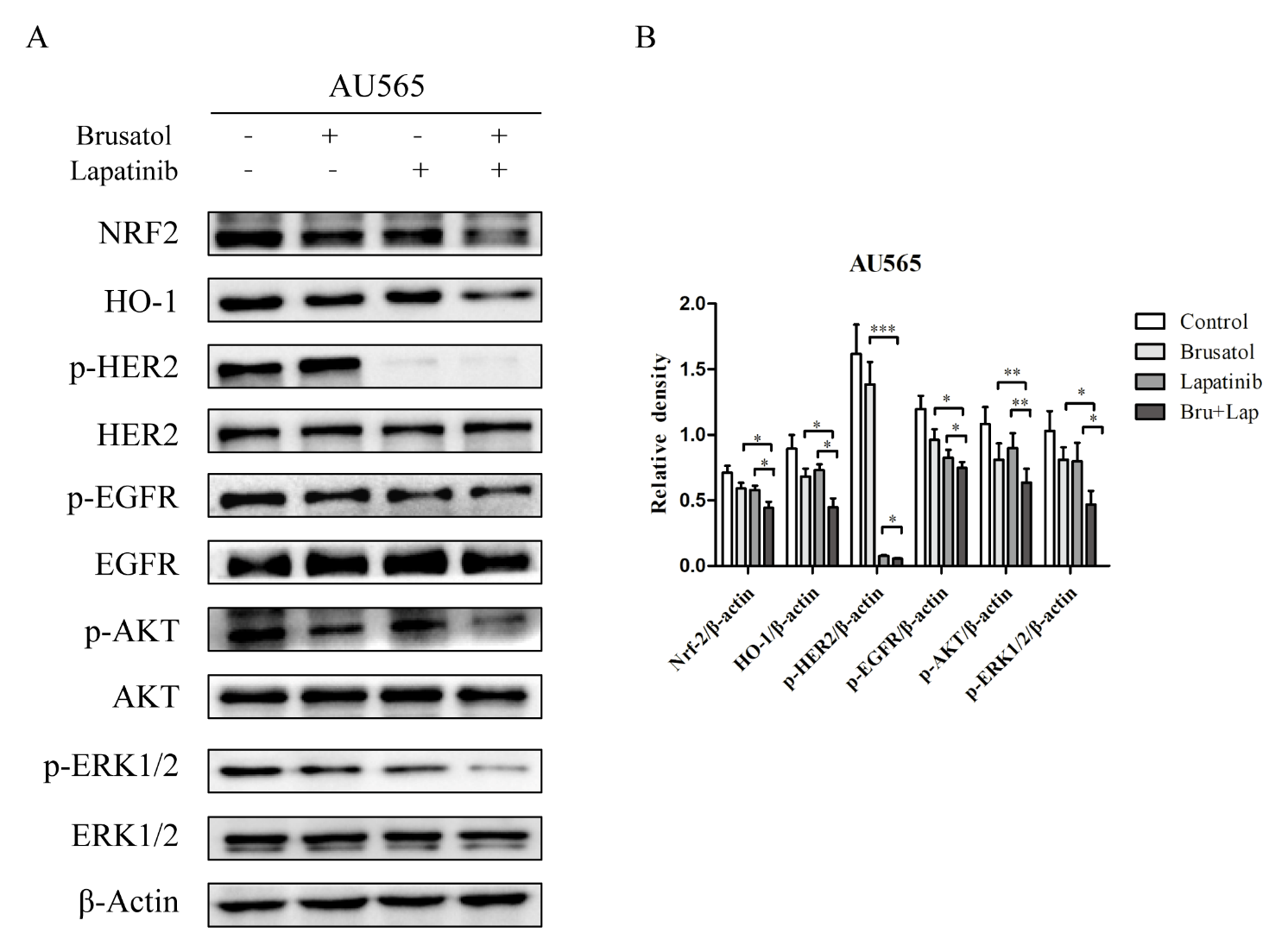


**Figure S4** SK-BR-3 and SK-OV-3 cells were treated with lapatinib or brusatol alone, or their combination for 24 hours. The changes in Nrf2/HO-1 and EGFR/HER2-AKT/ERK1/2 signaling pathways were monitored by Western Blotting.

**
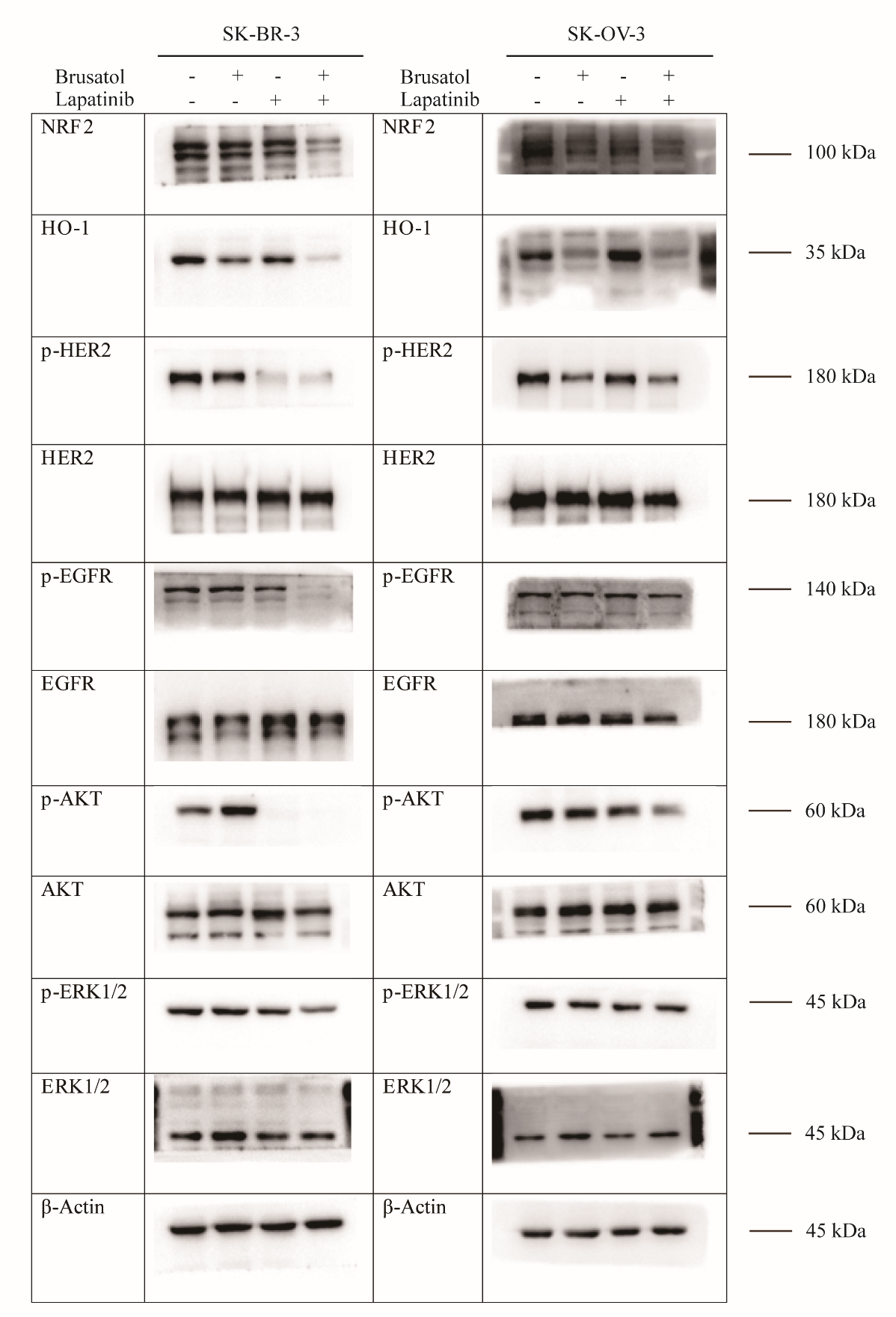
**

**Figure S5** Effect of Nrf2 knockdown on the expression of HO-1, p-HER2, p-AKT, and p-ERK1/2 were determined after treatment with Nrf2 siRNA or scramble siRNA for 36 h in SK-OV-3 cells by Western Blotting.


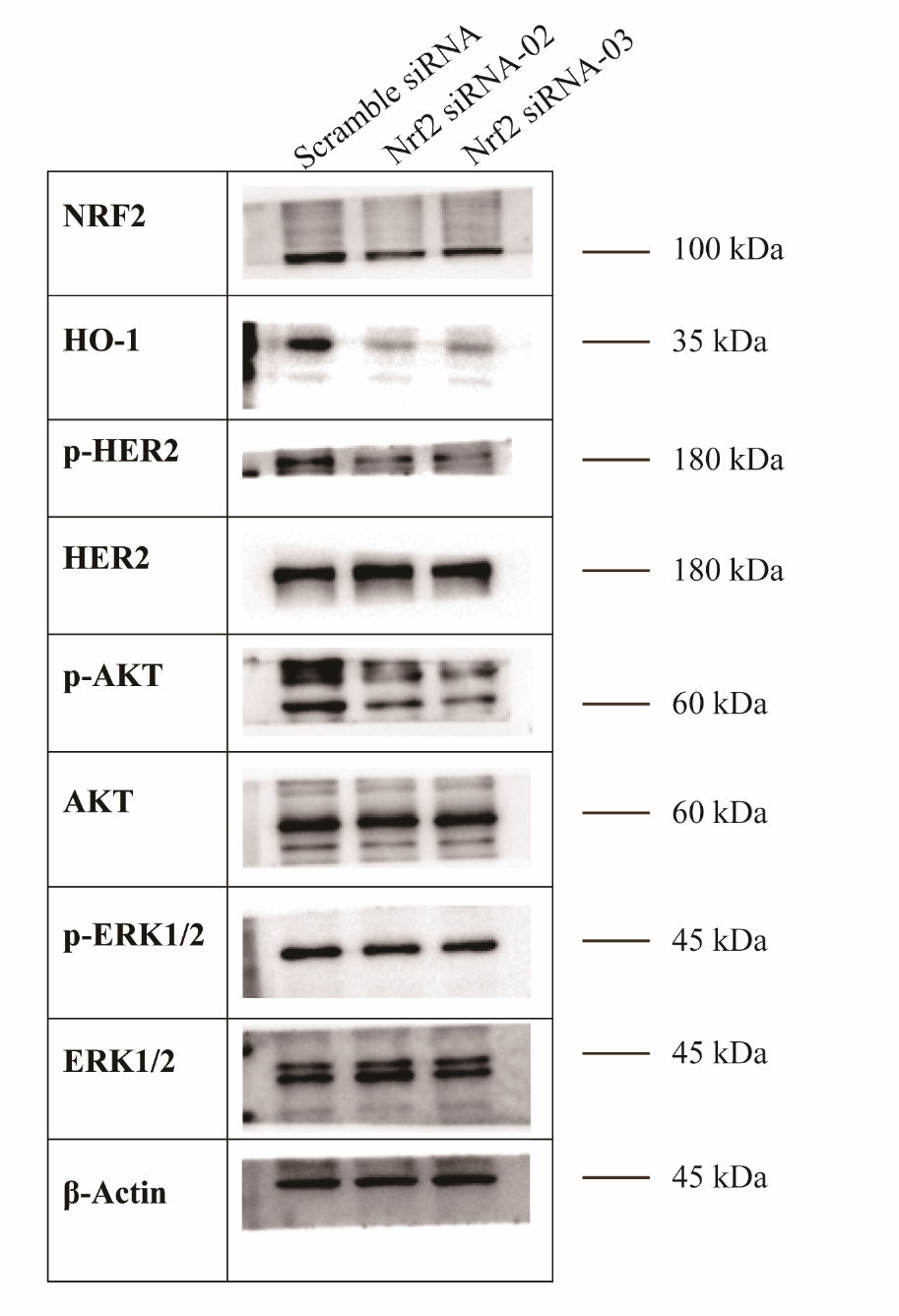


**Figure S6** Histological examination was conducted in SK-OV-3 tumor-bearing mice treated with lapatinib, brusatol or lapatinib plus brusatol. Representative images (magnification, ×200) of livers from nude mice (n=6) after injected with lapatinib, brusatol or lapatinib plus brusatol were obtained by staining with hematoxylin and eosin. Scale bars, 50 μm.


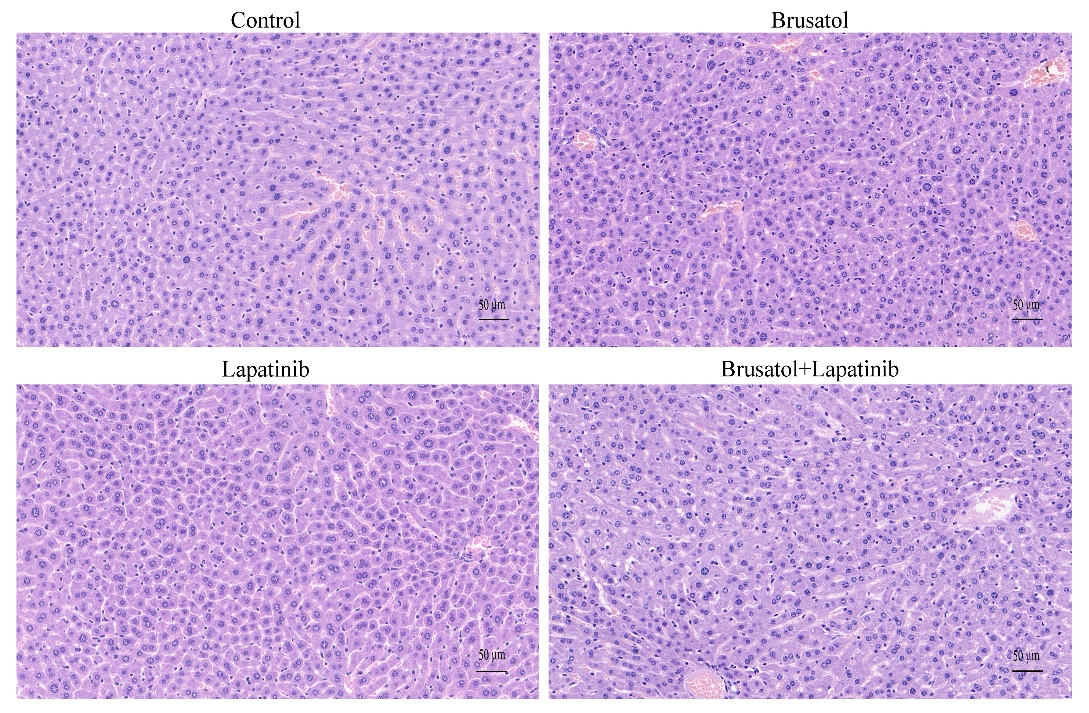


**Figure S****7 Inhibition of Nrf2 by brusatol for the indicated times.** SK-OV-3 cells were treated with brusatol in a time range from 0 to 30 hours. The level of Nrf2 was examined by Western Blotting.

**
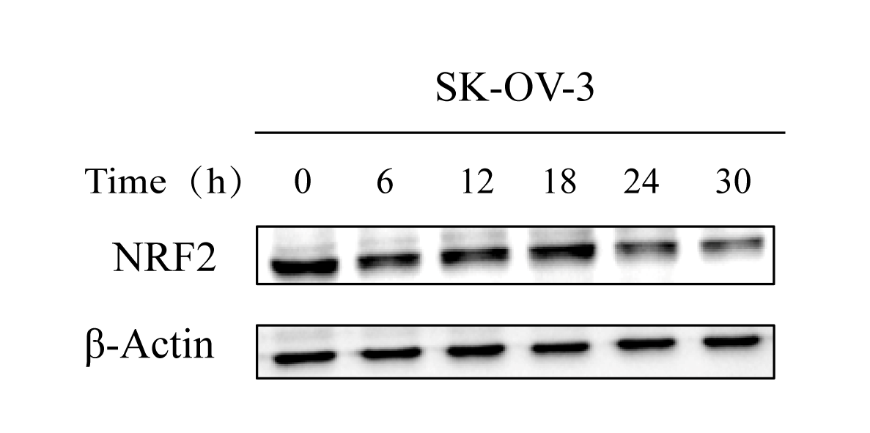
**
